# Effects of season of fire on bee‐flower interaction diversity in a fire‐maintained pine savanna

**DOI:** 10.1002/ece3.10450

**Published:** 2023-08-21

**Authors:** Michael Ulyshen, Kevin Robertson, Scott Horn, Cinnamon Dixon

**Affiliations:** ^1^ USDA Forest Service, Southern Research Station Athens Georgia USA; ^2^ Tall Timbers Research Station Tallahassee Florida USA

**Keywords:** Apoidea, forest, longleaf pine, phenological mismatch, seasonality, shortleaf pine

## Abstract

Whereas the Coastal Plain of the southeastern United States historically experienced fire primarily during the mid‐summer lightning season, managers today typically apply prescribed fire during the late winter or early spring months. The ecological implications of this discrepancy remain poorly understood, especially with regard to pollinators and their interactions with flowers. In a replicated field experiment, we compared the abundance and richness of bees and bee–flower interactions among pine savanna plots in Florida that were burned either during the winter, spring, summer, or fall. We netted 92 bee species from 77 species of flowers, representing 435 unique bee–flower interactions in total. When analyzing the results from each month separately, we detected significant short‐term reductions in the number of bees and bee–flower interactions following fires regardless of season. Although bee abundance and richness did not differ over the entire season, bee–flower interaction richness was significantly higher overall in spring and summer plots than in fall plots and the composition of both bees and bee–flower interactions differed significantly among treatments. Several bee–flower interactions were significantly associated with one or more of the treatments. Some of these associations could be attributed to differences in flowering phenology among treatments. Taken together, our findings suggest that season of fire has modest but potentially important implications for interactions between bees and flowers in southeastern pine ecosystems. Because most flowering plants within our study region are pollinated by a variety of bees and other insects, and most bees endemic to the region are polylectic, season of fire may not be very important to either group overall. However, the timing of fire may be more important to particular species including certain flower specialists and fire‐sensitive taxa such as butterflies. Future research targeting such species would be of interest.

## Graphical abstract

Season of fire (winter, spring, summer, or fall) did not affect the total richness or abundance of bees over the entire sampling period. However, the richness of bee–flower interactions was significantly higher in spring and summer plots than in fall plots and there were compositional differences among treatments. We conclude that season of fire has modest but potentially important implications for bees and their interactions with flowers in southeastern US pine savannas.

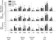

## 1 INTRODUCTION

Fire plays a key role in shaping the structure and composition of ecosystems throughout the world (McLauchlan et al., 2020). Historically, fires not started by humans resulted primarily from lightning strikes, with a region's productivity and climate influencing when and how often fires occurred as well as their severity, size, and horizontal spread (He et al., 2019). Land managers today increasingly rely upon prescribed fire to maintain desired plant assemblages and to mitigate wildfire risk. A guiding principle is that prescribed fire will have the greatest benefit when it most closely emulates the historic fire regime to which organisms are adapted. However, due to our limited understanding of historic fire regimes, experimental efforts are needed to determine the temporal patterns of fire that best sustain endemic fauna and flora. Here, we present the results from a study aimed at exploring the effects of season of fire on bee–flower interactions in southeastern US pine savannas.

Pine savannas and prairies that historically dominated the Coastal Plain of the southeastern United States prior to European colonization experienced some of the most frequent fires on the continent, with typical fire return intervals of 2 years or less (Guyette et al., 2012). Today, prescribed fire is required for maintaining these herb‐dominated communities representative of increasingly threatened “old‐growth grasslands” throughout the world (Bond & Parr, 2010; Krings et al., 2023; Veldman et al., 2015). Although there is evidence that fires across the region were historically concentrated during the late spring and summer months, coinciding with the period of greatest lightning activity, prescribed burns are now most commonly applied during the winter or spring months to moderate fire severity, take advantage of stronger and more directional winds, and to promote fire spread in less flammable post‐agricultural (partially restored) pine savannas (Palmer & Sisson, 2017; Ryan et al., 2013). Although season of fire is known to affect the growth, flowering phenology, and fruit production of certain plants within the region (Lewis & Harshbarger, 1976; Platt et al., 1988; Robertson & Hmielowski, 2014; Waldrop et al., 1992), only two previous studies, to our knowledge, have investigated how pollinators are affected by the timing of prescribed fire.

In a longleaf pine savanna in Georgia, Hiers et al. (2000) compared the effects of prescribed fire during the winter/early spring vs. the summer on the flowering phenology and fruit initiation (i.e., a surrogate for pollination rates) of legumes. They found season of fire to significantly alter flowering phenology but found no differences in fruit initiation between treatments. This latter finding, combined with the presence of bees capable of pollinating legumes during all flowering periods, suggests that pollinators and pollination may be largely unaffected by fire‐mediated differences in flowering phenology. Most recently, in Florida, Adedoja et al. (2022) found no significant differences in the number of pollinator visits observed on flowers among plots that had been burned during the “winter‐dry” (late January to mid‐March), “spring” (late May to mid‐June), or “summer‐wet” (August) periods. This was true both the year after the plots were burned and during the year of the burns. However, season of fire did affect flower density in that study, and these effects varied depending on time since fire. For example, summer‐wet burns reduced flower density during the year of the fire but increased the density of flowers the following year when no burns took place.

Both of these previous studies suggest that season of fire does not strongly affect pollinator abundance and visitation despite altering the phenology and density of flowers. However, little is known about species‐specific responses to season of fire. It is possible that some taxa are more sensitive to season of fire than others due to differences in activity periods and resource requirements among species. Most bee species native to the southeastern United States are polylectic (Folkerts et al., 1993), meaning they visit a wide range of host plants. However, some taxa are highly host specific and these species may be more sensitive to fire during particular times of year, especially if it results in a phenological mismatch between their activity and host–flower availability. Given limited knowledge of how season of fire affects the timing of flowering, and how this may differ among plant species (Brown et al., 2017), such phenological mismatches are difficult to predict. The current study sought to more fully explore such questions by investigating the effects of season of fire (winter, spring, summer, and fall) on specific bee–flower interactions in a Florida pine savanna.

## 2 METHODS

### 2.1 Study area and experimental design

**FIGURE 1:**
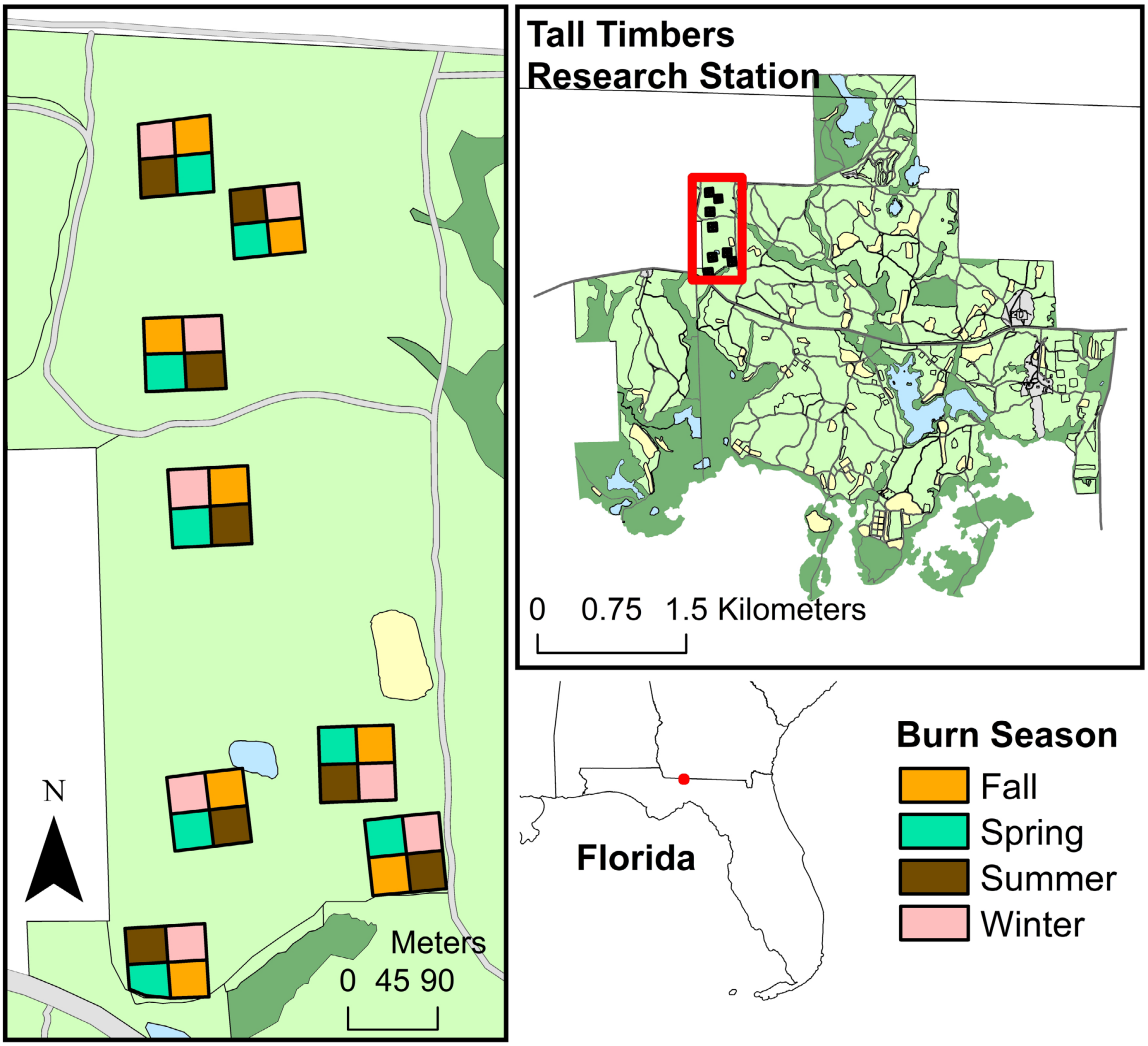
Map of study plots on Tall Timbers Research Station, Florida, USA. Light and dark green areas represent fire‐maintained open pine forests and unburned hardwood‐dominated forests, respectively.

This study took place at Tall Timbers Research Station in Leon County, Florida (Figure 1). The site had been fire‐excluded for some time prior to being acquired by Tall Timbers in 1990, after which broadleaf hardwood trees were cleared, leaving widely spaced shortleaf pine (*Pinus echinata Mill*.) in the overstory. Shortly after acquisition, eight 0.4 ha (one‐acre) plots, ultimately the experimental blocks used in the current study, were treated with various herbicides as a site‐preparation experiment and planted with longleaf pine. Today, the herbaceous plant community is indistinguishable from the surrounding vegetation, such that the herbicide had no noticeable long‐term effects (KR, personal observation). The study site has no history of agriculture and is dominated by little bluestem (*Schizachyrium scoparium (Michx*.*) Nash*) and resprouting native upland tree species top‐killed by fire, including mockernut hickory (*Carya tomentosa (Lam*. *ex Poir*.)), southern red oak (*Quercus falcata Michx*.), post oak (*Q*. *stellata Wangenh*.), white oak (*Q*. *alba* L.), and black oak (*Q*. *velutina Lam*.). Since acquisition by Tall Timbers, and prior to 2021 when the current treatments were initiated, the study site was treated with biennial prescribed burns in March–April. The three northern‐most and five southern‐most blocks used in this study were last burned this way in 2020 and 2019, respectively.

**FIGURE 2:**
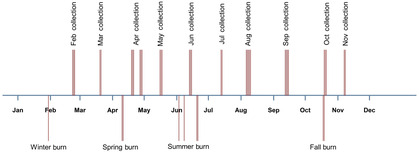
Timeline showing when bee collections and fires (above and below the line, respectively) took place in 2022. Note that multiple vertical lines reflect when collections or burns took place over multiple days. See text for specific dates, details about sampling effort by month, and for information on fires from previous years.

**FIGURE 3:**
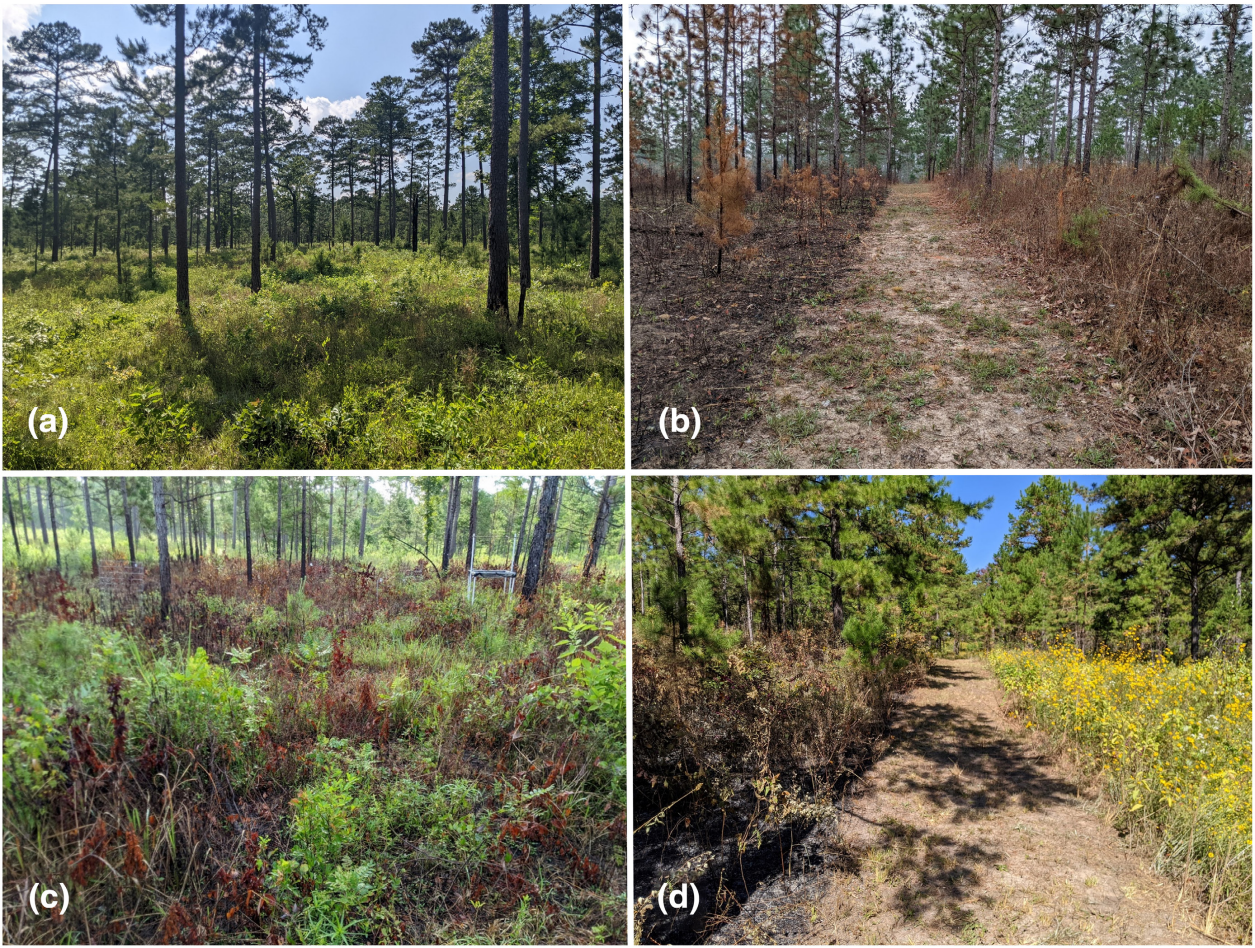
(a) View of the mature shortleaf pine savanna within which the study plots were embedded (May 2022). (b) Contrast between a recent winter burn (left) and a less‐recently burned plot (right; February 2022). (c) Example of a patchy summer burn (July 2022). (d) Contrast between a recent fall burn (left) and a less‐recently burned plot full of flowers (right; October 2022).

The current experiment followed a randomized complete block design consisting of the eight 0.4 ha blocks mentioned above. In January 2021, we subdivided each block into four 0.1 ha plots and randomly assigned them to one of four season of fire treatments, each to be conducted within a two‐month window: winter (December–January), spring (March–April), summer (June–July), and fall (September–October; Figure 1). In 2021, we burned the spring, summer, and fall plots on 4–13 April, 3–4 June, and 17–20 October, respectively (Note: there were no winter burns in 2021). In 2022, the year of pollinator sampling, we burned the winter, spring, summer, and fall burns on 30 January, 10–11 April, 3–21 June, and 18–19 October, respectively (Figure 2). Although we did not collect information on the horizontal spread of fire in 2022, we observed that the winter plots burned uniformly (Figure 3b) whereas some small unburned patches remained in most of the spring, summer, and fall plots (Figure 3c). Across treatments, fire behavior was comparable to that measured for prescribed fires 1 or 2 years following the previous fire in similar fuel types within 20 km of our study location (Robertson & Ostertag, 2007).

### 2.2 Bee sampling

Two of us (MDU and SH) sampled bees in each plot once a month from February to November 2022, with the first sampling occurring several weeks after the winter burn treatment (Figure 2). We used nets to sample bees from flowers, collecting only specimens judged to be actively foraging for pollen or nectar. Because we were specifically interested in bee–flower associations, bees flying near flowers or sitting on the leaves or petals of flowering plants were not collected. We identified flowers on site or from photographs with the help of botanists familiar with the local flora (KR and CD). The process for sampling bees followed a standardized protocol. Collectors 1 and 2 started in the southeast and northwest plot within each block, respectively, before moving to the next plot in the clockwise direction. Consequently, the two collectors never sampled the same plot at the same time. Within each plot, collectors 1 and 2 walked a diagonal transect from the southwest to northeast corners and from the northwest to southeast corners, respectively. All flowers present within a net length (~1.5 m) of either side of the transect were inspected for foraging bees. After completing the transect, the collectors walked the perimeter of each plot and then, as time allowed, searched for flowers elsewhere within the plot. Sampling took place only during sunny or partly cloudy weather between the hours of 9:30 am to 5:30 pm. We ensured that each collector sampled all plots within each block consecutively to minimize the effects of weather conditions and time of day on bee activity. Each collector spent 20 min (Feb, Mar, Aug, and Nov) or 25 min (Apr, May, Jun, Sep) sampling bees in each plot. In July, due to stormy weather, the plots within the four northern‐most blocks were each sampled for a total of 40 min while the others were sampled for only 30 min. In November, only one collector visited the plots. Thus, over the course of the season, we spent over 200 person‐hours sampling bees in the plots. Sampling took place on the following dates: February 22–24; March 20–21, April 19–29, May 16–18, June 13–15, July 13–14, August 6–10, September 12–15, October 19–21, and November 7–8 (Figure 2). Bees were identified to species using published (Gibbs, 2011; Mitchell, 1960, 1962) and online (discoverlife.org) keys as well as an established reference collection. Voucher specimens have been deposited in the first author's research collection.

### 2.3 Analysis

Unless otherwise stated, all analyses were conducted in R 4.2.1 (R Core Team, 2022). We pooled data by plot and month prior to analysis. Because only three of the summer plots had been burned prior to the June sampling, we included only the five unburned plots in the analysis for that month. We calculated the total bee–flower interaction richness, bee richness, and bee abundance for each plot by month. We calculated the same metrics after combining data across months. These monthly and combined responses were compared among treatments using generalized linear mixed effects models with treatment as the fixed effect and block as the random term. We used Poisson models for bee–flower interaction richness and bee richness and negative binomial models for bee abundance to mitigate overdispersion (identified using the dispersion_glmer function). For multiple comparisons of season of fire treatments, we used the ghlt function of the multcomp package (Hothorn et al., 2016). Because the winter plots yielded no bees in February, the Kruskal–Wallis rank sum test was used to compare treatments for that month. Pairwise comparisons were made using the Benjamini–Hochberg method to adjust *p*‐values.

To test whether bees and bee–flower interactions differed compositionally among the season of fire treatments, we conducted non‐metric multidimensional scaling followed by PERMANOVA in PC‐ORD (McCune & Mefford, 2011). We pooled data across sampling periods when constructing the matrices. Then, to test which bee species or bee–flower interactions were strongly associated with one or more season of fire treatments, we conducted indicator species analysis using the function multipatt (multilevel pattern analysis) in the package indicspecies (Cáceres & Legendre, 2009) to produce indicator values ranging from 0 (no association) to 1 (complete association).

Finally, to estimate the total bee richness at our study site, we calculated the Chao1 estimator using the rareNMtests package (Cayuela & Gotelli, 2014) which gives a richness estimate with 95% confidence intervals based on the list of collected species and their abundances.

## 3 RESULTS

In total we collected 92 bee species (Appendix A) from 77 flower species (Appendix B) and recorded 435 unique interactions between bee species and flower species. The five bee taxa with the greatest observed host range were *Augochloropsis metallica* (Fabricius), *Lasioglossum reticulatum* (Robertson), *Ceratina* sp., *Lasioglossum apopkense* (Robertson), and *Bombus impatiens* Cresson (Table 1). The five plant species yielding the greatest richness of bees were *Helianthus angustifolius* L., *Pityopsis aspera* (Shuttlw. ex Small) Small, *Chrysopsis mariana* (L.) Elliot, *Rubus cuneifolius* Pursh, and *Tephrosia virginiana* (L.) Pers. (Table 1). Based on the Chao1 estimator for bee richness (estimate: 114 species, 95% confidence interval: 99.5–156.3), we likely collected over 80% of the bee species present at our study site.

**TABLE 1:** Bee taxa with the widest observed host range in this study ranked by the number of flower records (left) and flower taxa ranked by the number of collected bee species (right).

Rank	Bee	No. flower records	Plant	No. bee species
1	*Augochloropsis metallica* (Fabricius)	35	*Helianthus angustifolius* L.	23
2	*Lasioglossum reticulatum* (Robertson)	31	*Pityopsis aspera* (Shuttlw. ex Small) Small	23
3	*Ceratina* sp.	26	*Chrysopsis mariana* (L.) Elliot	21
4	*Lasioglossum apopkense* (Robertson)	19	*Rubus cuneifolius* Pursh	21
5	*Bombus impatiens* Cresson	18	*Tephrosia virginiana* (L.) Pers.	21
6	*Augochloropsis sumptuosa* (Smith)	16	*Baptisia alba* (L.) Vent.	17
7	*Lasioglossum weemsi/leviense*	14	*Silphium asteriscus* L.	15
8	*Megachile petulans* Cresson	14	*Mimosa quadrivalvis* L.	13
9	*Agapostemon splendens* (Lepeletier)	13	*Polygala polygama* Walter	12
10	*Megachile mendica* Cresson	13	*Solidago altissima* L.	10

**FIGURE 4:**
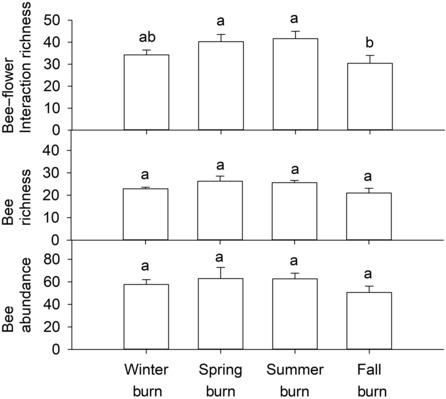
Mean ± SE (*n* = 8) interaction richness (top), bee richness (middle), and bee abundance (bottom) by season of fire after combining data across sampling periods. Within each graph, bars with different letters above them are significantly different.

**FIGURE 5:**
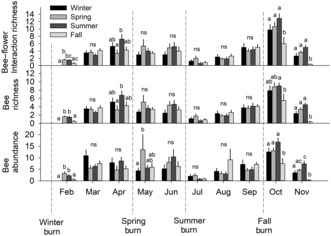
Mean ± SE interaction richness (top), bee richness (middle), and bee abundance (bottom) by month (2022) and season of fire (*n* = 8 except for summer plots in June for which *n* = 5). For each month, bars with different letters are significantly different. Non‐significant differences are indicated by “ns.” Vertical dashed lines indicate the approximate timing of the four season of fire treatments.

After combining data across sampling periods, we found no significant differences in the richness or abundance of bees among season of fire treatments (Figure 4). However, there were, on average, about 10–11 fewer bee–flower interactions detected in fall plots than in spring or summer plots, a significant difference (Figure 4). When we analyzed data from each month separately, we detected several significant differences between treatments. In February, following winter burns, for instance, we found lower bee–flower interaction richness, bee richness, and bee abundance in the fall and especially winter plots compared to the spring and summer plots (Figure 5). However, no such differences were observed 1 month later. In April, after the spring burns, both bee–flower interaction and bee richness were significantly lower in spring than summer plots but there were no significant differences between the spring and fall or winter plots. In May, we found bee abundance to be significantly higher in the spring plots than in the winter plots. Otherwise, there were no other significant differences among treatments until October, following the fall burns, when bee–flower interaction richness, bee richness, and bee abundance were all significantly lower in the fall plots than in the other treatments. The same pattern was detected in November.

**FIGURE 6:**
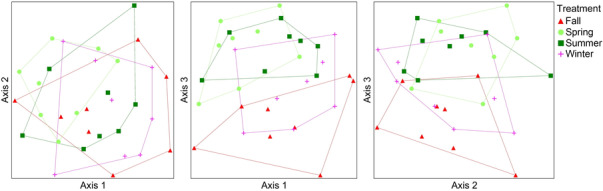
NMDS ordination from three perspectives showing compositional differences in bee–flower interactions among the four season of fire treatments across the entire season.

NMDS based on bee data yielded a one‐dimensional solution (not shown). PERMANOVA revealed significant compositional differences in bee communities among the season of fire treatments (*F* = 1.42, *p* = .01). Pairwise comparisons revealed significant differences between fall and spring plots (*t* = 1.30, *p* = .03) and between fall and summer plots (*t* = 1.26, *p* = .04). According to indicspecies analysis, four bee species were significantly associated with one of the fire seasons: *Melissodes tepaneca* Cresson with the fall plots (IV = 0.61, *p* = .05), *Megachile texana* Cresson with the spring plots (IV = 0.78, *p* < .01), *Pseudopanurgus labrosiformis* Cresson with the summer plots (IV = 0.69, *p* = .02), and *Megachile exilis* Cresson with the winter plots (IV = 0.68, *p* = .03). NMDS based on bee–flower interactions yielded a three‐dimensional solution with a final stress of 18.4. The ordination shows distinct separation between bee–flower interactions in spring and summer plots and those in fall plots (Figure 6). PERMANOVA confirmed significant compositional differences in bee–flower interactions among the treatments (*F* = 1.58, *p* < .001). Pairwise comparisons revealed significant differences among all treatment pairs except for the fall and winter treatments (Table 2). A total of 10 bee–flower interactions were found to be significantly associated with one or more treatments based on indicspecies analysis (Table 3). Six of these associations involved spring or summer plots while four involved fall or winter plots (Table 3).

**TABLE 2:** Results from PERMANOVA showing pairwise comparisons of bee–flower interactions among season of fire treatments.

Comparison	*t*	*p*
Fall vs. spring	1.358	.0004
Fall vs. summer	1.2556	.0082
Fall vs. winter	1.1063	.1112
Spring vs. summer	1.1934	.0242
Spring vs. winter	1.3351	.0036
Summer vs. winter	1.2781	.0088

**TABLE 3:** Results from indicspecies showing significant associations between interactions and one or more of the season of fire treatments.

Interaction		Spring	Summer	Fall	Winter	Statistic
Bee	Plant					
*Megachile petulans* Cresson	*Tephrosia virginiana* (L.) Pers.			x		IV = 0.85, *p* = .001
*Anthidiellum notatum* (Latreille)	*Tephrosia virginiana* (L.) Pers.	x				IV = 0.772, *p* = .004
*Lasioglossum reticulatum* (Robertson)	*Gelsemium sempervirens* (L.) J. St.‐Hil.	x				IV = 0.745, *p* = .002
*Lasioglossum reticulatum* (Robertson)	*Tephrosia virginiana* (L.) Pers.	x				IV = 0.693, *p* = .022
*Megachile mendica* Cresson	*Rubus cuneifolius* Pursh		x			IV = 0.791, *p* = .001
*Pseudopanurgus labrosiformis* Cresson	*Pityopsis aspera* (Shuttlw. ex Small) Small		x			IV = 0.707, *p* = .008
*Megachile exilis* Cresson	*Tephrosia virginiana* (L.) Pers.				x	IV = 0.745, *p* = .009
*Habropoda laboriosa* (Fabricius)	*Baptisia alba* (L.) Vent.			x	x	IV = 0.768, *p* = .014
*Augochloropsis metallica* (Fabricius)	*Solidago arguta* Aiton			x	x	IV = 0.715, *p* = .02
*Habropoda laboriosa* (Fabricius)	*Gelsemium sempervirens* (L.) J. St.‐Hil.	x	x			IV = 0.829, *p* = .001

## 4 DISCUSSION

The purpose of this study was to better understand how season of fire affects bees and bee–flower interactions in pine savannas on the southeastern US Coastal Plain. We found no difference in the total richness or abundance of bees among treatments across the entire season. However, bee–flower interaction richness was significantly lower in fall plots than in spring or summer plots and we detected significant differences in the composition of both bees and bee–flower interactions among treatments.

We observed stronger effects of season of fire when we analyzed data from each month separately. Results from February, April, October, and November all confirm that fire greatly reduces or eliminates floral resource availability and associated pollinator activity. However, these effects appear to be quite ephemeral, disappearing within 1–2 months due to the rapid recovery of plants following a fire. We even found evidence that fire can trigger flowering by some plant species with resultant increases in bee numbers relative to less‐recently burned plots. In May, for example, just 5 weeks after the spring burns, we found bee abundance to be significantly higher in spring plots than in winter plots. This was driven by a fire‐induced flush of *Tephrosia virginiana* from which we collected large numbers of bees.

According to indicator species analysis, all four seasons of fire promoted particular bee–flower interactions. This was driven primarily by the effects of season of fire on the flowering phenology of different plant species. Particularly good examples of this are provided by the flowering time of *Tephrosia virginiana*. Whereas *T*. *virginiana* bloomed in April in the absence of fire, the burning of the spring plots in April resulted in a several week delay in flowering by the species. This largely explains why the interaction between *Megachile exilis* and *T*. *virginiana* was significantly associated with winter plots and not with spring plots, given that *M*. *exilis* was much more active in April (74% of records) than in May. However, it is not clear why this association was more strongly associated with winter plots than summer or fall plots. Similarly, the profusion of *T*. *virginiana* blooms in the spring plots in May likely explains why two bee taxa were strongly associated with *T*. *virginiana* in the spring plots.

While our findings show that season of fire can influence bee–flower interactions by affecting the phenology of flowering, this does not appear to be very ecologically significant in our system. Most bee species within our study area are polylectic (Folkerts et al., 1993), meaning they collect pollen from a wide variety of unrelated plants. Similarly, many of the flower species we sampled were visited by numerous species of bees in this study. Thus, fire‐driven phenological mismatches between bees and flowers are probably largely inconsequential for both parties in this region. Not one of the species pairs found to be significantly associated with one or more of the seasons of fire in this study consists of species belonging to specialist bee–flower relationships. However, as discussed below, we should not be too hasty in concluding that season of fire is unimportant to pollinators in southeastern US pine savannas.

It should be stressed that because the spring, summer, and fall burns did not all cover 100% of the plots, flowers were often available in these plots even immediately after burns. We would likely have detected stronger effects of season of fire if these plots had burned more uniformly. A second factor likely to have diminished our ability to detect the effects of season of fire concerns plot size. Typical burn units within our region are often more than three orders of magnitude larger than the 0.25 ha plots sampled in this study. Because bees could so rapidly colonize our plots from the surrounding matrix, our results may underestimate the effects season of fire may have on bees in larger burn units. Considering that floral resource availability can be greatly reduced for several weeks following a fire, and the foraging lives of bees are often limited to just a few weeks (Danforth et al., 2019), season of fire may be more important when large areas are burned compared to the small plots we studied. Although the studied community type is composed of mostly perennial plants that resprout after being top‐killed by fire, it is possible that season of fire may gradually change plant species composition, and potentially pollinator communities, over time through changes in flowering phenology and reproductive success. However, studies in similar perennial grasslands and savannas find such changes to occur over multiple decades and involve mostly changes in species relative abundance rather than species composition (Glitzenstein, 2003; Smith et al., 2013; Towne & Owensby, 1984; Van Wyk, 1971). Finally, it is possible that season of fire has a stronger effect on taxa not considered here. Butterflies, for example, are generally more exposed to fire during their immature stages because of their use of plant hosts and are more sensitive to fire as a result (Carbone et al., 2019). Season of fire has important implications for some of these species (Jue et al., 2022).

A particularly unique aspect of this study is the inclusion of fall fires, which are barely represented in the fire ecology literature pertaining to southeastern US pine savannas (Lewis, 1964; Platt et al., 1988). Although the early October burns were within the growing season, we observed little to no regrowth of perennial vegetation in burned areas until the following growing season, consistent with observations of growing season fall burning in a tallgrass prairie (Engle et al., 2000). As a consequence, bee visitation and flowering phenology were quite similar between the winter burn and fall burn plots at the beginning of the growing season, as if both had been recently burned, similar to studies in other pine savannas (Lewis, 1964; Platt et al., 1988). Bees recorded in the fall plots in October and November were captured from patches of unburned vegetation rather than from vegetation recovering from the fire. This contrasts with the rapid recovery of vegetation observed following spring and summer burns and likely explains why bee–flower interactions were significantly lower in the fall plots than in those other treatments. Although observed differences among treatments were small, our findings suggest that fall burns may be the least favorable to bee–flower interactions, especially considering that fall is the season of greatest floral availability in our study area (Figure 3d).

Finally, we should address an important limitation inherent to season of fire studies. This concerns the fact that season of fire and time since fire are unavoidably confounded. For example, comparisons of October bee data among winter, spring, summer, and fall plots cannot isolate the effects of season of fire when those plots were last burned about nine, six, four, and zero months previously. Because pollinators and the blooming periods of plants are both highly seasonal, this problem cannot be overcome simply by limiting comparisons among season of fire treatments to the same time since fire. Instead, we chose to combine data across months. Because all treatments (except for the winter burns) were implemented in 2021, the year before sampling took place, the average time since the last burn over the entire sampling period would be roughly the same across treatments. We do not consider the fact that winter plots were not burned in 2021 to be too problematic since they were burned at the very beginning of 2022 and so at no point was a plot sampled more than 1 year since the last burn. Thus, these combined results reflect the effects of season of fire over the entire year in terms relevant to managers interested in conserving plants, pollinators, and the interactions between them. The results from the monthly analyses should be interpreted with more caution. However, we feel they are valuable in better understanding the immediate and delayed effects of burns conducted at different times of the year.

In conclusion, our findings suggest that season of fire has modest but potentially important implications for interactions between bees and flowers in southeastern pine ecosystems. Despite the weak effects reported here, incorporating a variety of fire seasons into management plans has the potential to enhance habitat heterogeneity across the landscape in ways that can benefit bees and other pollinators. For example, burning at different times of the year can help stagger floral resource availability (Hiers et al., 2000) thereby enhancing pyrodiversity and pollinator diversity across the landscape (Ponisio et al., 2016; Ulyshen et al., 2022). Future work targeting butterflies or other sensitive taxa would be of great interest.

## AUTHOR CONTRIBUTIONS

**Michael Ulyshen:** Conceptualization (equal); data curation (lead); formal analysis (lead); investigation (lead); methodology (lead); writing – original draft (lead). **Kevin Robertson:** Conceptualization (equal); formal analysis (supporting); investigation (equal); methodology (supporting); writing – original draft (supporting). **Scott Horn:** Investigation (supporting); writing – original draft (supporting). **Cinnamon Dixon:** Investigation (supporting); writing – original draft (supporting).

## CONFLICT OF INTEREST STATEMENT

The authors declare no conflict of interest.

## Data Availability

The dataset produced by this study is available at https://doi.org/10.5061/dryad.x95x69pqr.

## APPENDIX A

### A.1

List of bee species collected in this study. With the exception of *Apis mellifera*, numbers reflect the total abundance of each species by month (left columns) and treatment (right columns). Data for *A*. *mellifera* represent the sum of flower records across plots as it was not possible to fully capture the abundance of this species at certain times of the year.

**Table:**

Species	Month										Treatment				Total
	Feb	Mar	Apr	May	Jun	Jul	Aug	Sep	Oct	Nov	Winter	Spring	Summer	Fall	
*Agapostemon sericeus* (Forster)	0	0	1	0	0	0	0	0	0	0	0	1	0	0	1
*Agapostemon splendens* (Lepeletier)	0	2	3	0	4	0	0	0	11	10	13	10	4	3	30
*Agapostemon virescens* (Fabricius)	0	0	0	0	1	0	0	0	0	0	0	1	0	0	1
*Andrena accepta* Viereck	0	0	0	0	0	0	0	0	17	0	4	4	5	4	17
*Andrena fulvipennis* (Smith)	0	0	0	0	0	0	0	0	2	3	0	1	3	1	5
*Andrena hilaris* Smith	0	12	0	0	0	0	0	0	0	0	0	6	5	1	12
*Anthidiellum notatum* (Latreille)	0	0	1	25	2	0	2	3	0	0	7	22	1	3	33
*Anthidiellum perplexum* (Smith)	0	0	2	2	0	0	0	0	0	0	1	2	0	1	4
*Apis mellifera* L.	0	0	0	0	0	1	0	2	54	0	14	14	18	11	57
*Augochlorella aurata* (Smith)	0	1	8	3	1	0	2	0	1	8	6	7	7	4	24
*Augochloropsis anonyma* (Cockerell)	0	0	3	1	1	0	1	0	0	0	0	2	3	1	6
*Augochloropsis metallica* (Fabricius)	0	11	22	8	51	1	48	54	41	14	58	40	70	82	250
*Augochloropsis sumptuosa* (Smith)	0	0	16	1	4	0	0	1	11	8	4	11	19	7	41
*Bombus bimaculatus* Cresson	0	0	0	0	0	0	0	0	15	0	8	2	5	0	15
*Bombus fraternus* (Smith)	0	0	0	0	0	0	0	1	0	0	0	1	0	0	1
*Bombus griseocollis* (De Geer)	0	3	4	0	0	0	0	1	0	0	3	2	1	2	8
*Bombus impatiens* Cresson	2	12	2	1	0	0	0	3	24	0	9	13	14	8	44
*Bombus pensylvanicus* (De Geer)	0	0	1	2	0	0	1	1	0	0	0	3	1	1	5
*Caupolicana electa* (Cresson)	0	0	0	0	0	0	0	0	2	0	0	0	2	0	2
*Ceratina* sp.	5	8	25	10	21	1	1	1	0	2	11	14	22	27	74
*Coelioxys mexicanus* Cresson	0	0	0	0	0	0	0	2	0	0	0	2	0	0	2
*Coelioxys sayi* Robertson	0	0	1	0	0	0	0	2	0	0	1	1	1	0	3
*Colletes brevicornis* Robertson	0	0	1	0	0	0	0	0	0	0	0	0	0	1	1
*Colletes nudus* Robertson	0	0	0	0	0	0	2	0	0	0	1	0	0	1	2
*Colletes thysanellae* Mitchell	0	0	0	0	0	0	0	0	1	0	0	0	1	0	1
*Dieunomia heteropoda* (Say)	0	0	0	0	0	0	0	0	1	0	0	0	1	0	1
*Epeolus lectoides* Robertson	0	0	0	0	0	0	0	0	1	0	0	0	1	0	1
*Eucera dubitata* (Cresson)	0	126	3	0	0	0	0	0	0	0	54	17	27	31	129
*Habropoda laboriosa* (Fabricius)	26	35	1	0	0	0	0	0	0	0	13	18	18	13	62
*Halictus poeyi/ligatus*	0	0	21	6	21	3	0	2	9	27	26	22	24	17	89
*Heriades variolosa/leavitti*	0	0	5	2	0	0	0	3	0	0	1	3	4	2	10
*Hoplitis pilosifrons* (Cresson)	0	0	2	0	0	0	0	0	0	0	0	1	0	1	2
*Hoplitis truncata* (Cresson)	0	0	9	3	0	0	0	0	0	0	7	5	0	0	12
*Hylaeus floridanus* (Robertson)	0	0	0	0	0	0	0	0	1	0	1	0	0	0	1
*Lasioglossum apopkense* (Robertson)	0	0	0	14	26	2	11	17	3	1	16	25	16	17	74
*Lasioglossum creberrimum* (Smith)	1	0	0	0	0	0	0	0	0	0	0	0	1	0	1
*Lasioglossum floridanum* (Robertson)	0	0	1	0	0	0	0	0	0	0	1	0	0	0	1
*Lasioglossum hitchensi* Gibbs	0	0	0	1	0	0	0	0	0	0	0	1	0	0	1
*Lasioglossum illinoense* (Robertson)	0	0	0	0	0	0	0	2	1	0	1	1	0	1	3
*Lasioglossum imitatum* (Smith)	0	0	0	0	6	0	3	1	1	1	2	3	6	1	12
*Lasioglossum longifrons* (Baker)	0	0	0	1	1	0	0	0	0	0	1	0	1	0	2
*Lasioglossum pectorale* (Smith)	0	3	11	6	0	1	1	0	10	14	6	15	16	9	46
*Lasioglossum reticulatum* (Robertson)	10	9	9	53	34	8	44	34	11	0	42	69	35	66	212
*Lasioglossum tegulare/puteulanum*	0	3	4	1	1	0	0	0	0	0	1	0	2	6	9
*Lasioglossum trigeminum* Gibbs	0	1	0	1	0	0	0	0	0	0	1	0	0	1	2
*Lasioglossum vierecki* (Crawford)	0	1	0	2	1	0	0	0	0	0	1	1	1	1	4
*Lasioglossum weemsi/leviense*	1	3	8	4	6	6	4	3	0	0	13	4	11	7	35
*Megachile addenda* Cresson	0	0	0	1	0	0	0	0	0	0	0	1	0	0	1
*Megachile albitarsis* Cresson	0	0	0	7	3	0	3	5	0	0	4	6	5	3	18
*Megachile campanulae* (Robertson)	0	0	0	2	0	0	0	0	0	0	0	2	0	0	2
*Megachile deflexa* Cresson	0	0	0	0	0	0	1	0	0	0	0	0	0	1	1
*Megachile exilis* Cresson	0	0	17	4	2	0	0	0	0	0	17	4	2	0	23
*Megachile frugalis* Cresson	0	0	0	2	0	0	0	0	0	0	0	2	0	0	2
*Megachile georgica* Cresson	0	0	5	8	1	4	0	1	0	0	4	9	4	2	19
*Megachile inimica* Cresson	0	0	0	0	0	0	0	1	0	0	0	0	1	0	1
*Megachile mendica* Cresson	0	0	9	0	0	6	0	8	6	1	8	8	12	2	30
*Megachile mucida* Cresson	0	1	0	0	0	0	0	0	0	0	0	1	0	0	1
*Megachile parallela* Smith	0	0	0	0	0	0	1	0	1	0	1	0	0	1	2
*Megachile petulans* Cresson	0	0	3	34	4	7	0	10	0	0	7	37	8	6	58
*Megachile pseudobrevis* Mitchell	0	0	4	2	0	0	0	9	3	0	5	5	4	4	18
*Megachile texana* Cresson	0	0	0	3	1	4	0	3	0	0	0	9	0	2	11
*Megachile xylocopoides* Smith	0	0	0	0	0	0	1	1	0	0	1	1	0	0	2
*Melissodes bimaculatus* (Lepeletier)	0	0	0	0	0	0	0	2	0	0	0	0	0	2	2
*Melissodes boltoniae* Robertson	0	0	0	0	0	0	0	2	27	1	2	5	20	3	30
*Melissodes communis* Cresson	0	0	0	3	3	0	0	0	0	0	0	2	1	3	6
*Melissodes comptoides* Robertson	0	0	0	0	0	0	3	1	0	0	1	1	2	0	4
*Melissodes dentiventris* Smith	0	0	0	0	0	0	0	0	32	0	10	10	7	5	32
*Melissodes druriellus* (Kirby)	0	0	0	0	0	0	0	0	54	0	15	14	20	5	54
*Melissodes manipularis* Smith	0	0	0	0	0	0	0	0	1	0	0	1	0	0	1
*Melissodes tepaneca* Cresson	0	0	0	0	3	0	0	0	0	0	0	0	0	3	3
*Melissodes trinodis* Robertson	0	0	0	0	0	0	0	0	1	0	0	0	0	1	1
*Melitoma taurea* (Say)	0	0	0	22	13	4	7	0	0	0	12	7	21	6	46
*Melitta americana* (Smith)	0	0	2	0	0	0	0	0	0	0	0	0	2	0	2
*Nomada affabilis* Cresson	0	0	1	0	0	0	0	0	0	0	0	0	1	0	1
*Nomia nortoni* Cresson	0	0	0	0	0	0	1	1	1	0	1	1	1	0	3
*Osmia inspergens* Lovell and Cockerell	0	3	2	0	0	0	0	0	0	0	0	2	1	2	5
*Osmia sandhouseae* Mitchell	1	7	2	0	0	0	0	0	0	0	4	1	1	4	10
*Paranthidium jugatorium* (Say)	0	0	0	0	0	0	0	3	15	22	10	9	14	7	40
*Perdita bequaerti* Viereck	0	0	0	0	0	0	0	0	2	1	1	0	2	0	3
*Perdita bishoppi* Cockerell	0	0	0	0	0	0	0	4	11	9	6	5	10	3	24
*Pseudopanurgus aestivalis* Crawford	0	0	0	0	0	0	0	1	0	0	0	0	1	0	1
*Pseudopanurgus labrosiformis* Cresson	0	0	0	0	0	0	0	0	7	1	1	1	6	0	8
*Pseudopanurgus rugosus* Robertson	0	0	0	0	0	0	0	0	10	3	3	6	3	1	13
*Pseudopanurgus solidaginus* Robertson	0	0	0	0	0	0	0	0	9	1	4	4	1	1	10
*Ptilothrix bombiformis* (Cresson)	0	0	0	0	1	0	3	0	0	0	1	0	3	0	4
*Sphecodes fattigi* Mitchell	0	0	1	0	0	0	0	0	0	0	1	0	0	0	1
*Stelis louisae* Cockerell	0	0	0	0	0	0	0	1	0	0	0	0	0	1	1
*Svastra aegis* LaBerge	0	0	0	0	0	0	5	0	0	1	3	1	0	2	6
*Svastra atripes* (Cresson)	0	0	0	0	0	0	10	3	0	0	10	1	0	2	13
*Tetraloniella cressoniana* Cockerell?	0	0	0	0	0	0	0	0	1	0	1	0	0	0	1
*Trachusa ridingsii* (Cresson)	0	0	0	0	0	0	0	1	0	0	0	0	1	0	1
*Xylocopa virginica* (Linnaeus)	3	0	2	0	1	0	0	0	0	0	1	3	1	1	6
Total abundance	49	241	212	235	213	48	155	190	398	128	461	503	501	404	1869
Total number of species	8	18	36	32	26	13	22	36	36	19	58	65	61	57	92

## APPENDIX B

### B.1

List of flower species from which bees were collected in this study. Data reflect the total number of bees collected from each species by month.

**Table:**

Species	Feb	Mar	Apr	May	Jun	Jul	Aug	Sep	Oct	Nov	Total
*Agalinis fasciculata* (Elliott)	0	0	0	0	0	0	0	1	7	0	8
*Ageratina aromatica* (L.)	0	0	0	0	0	0	0	0	13	9	22
*Amianthium muscitoxicum* (Walter) A. Gray	0	0	1	0	0	0	0	0	0	0	1
*Asclepias tuberosa* L.	0	0	0	6	13	1	0	0	0	0	20
*Asclepias variegata* L.	0	0	1	0	0	0	0	0	0	0	1
*Aureolaria flava* (L.) Farw.	0	0	0	0	0	0	0	4	0	0	4
*Baptisia alba* (L.) Vent.	0	179	18	2	0	0	0	0	0	0	199
*Callicarpa americana* L.	0	0	0	0	75	1	0	0	0	0	76
*Centrosema virginianum* (L.) Benth.	0	0	0	0	2	0	0	0	0	0	2
*Ceonothus americanus* L.	0	0	1	2	0	0	0	0	0	0	3
*Chamaecrista fasciculata* (Michx.) Greene	0	0	0	0	0	0	3	9	0	0	12
*Chamaecrista nictitans* (L.) Moench	0	0	0	0	0	0	0	24	0	0	24
*Chrysopsis mariana* (L.) Elliot	0	0	0	3	0	0	0	0	94	57	154
*Cirsium horridulum* Michx.	0	4	0	0	0	0	0	0	0	0	4
*Cnidoscolus urens var*. *stimulosus* (Michx.) Govaerts	0	0	3	0	0	0	0	0	0	0	3
*Conoclinium coelestinum* (L.) DC.	0	0	0	0	0	0	0	2	1	0	3
*Conyza canadensis* (L.) Cronquist	0	0	0	0	0	0	0	0	0	1	1
*Crocanthemum carolinianum* (Walter) Spach.	1	4	0	0	0	0	0	0	0	0	5
*Crotalaria lanceolata* E. Mey.	0	0	0	0	0	0	0	1	0	0	1
*Crotalaria rotundifolia* J.F. Gmel.	0	1	0	0	0	0	0	0	0	0	1
*Crotalaria spectabilis* Roth	0	0	0	0	0	0	0	1	0	0	1
*Cuscuta campestris* Yunck.	0	0	0	0	0	0	0	0	0	5	5
*Desmodium laevigatum* (Nutt.) DC.	0	0	0	0	0	0	0	4	0	0	4
*Dyschoriste oblongifolia* (Michx.) Kuntze	0	0	9	1	0	0	0	0	0	0	10
*Elephantopus elatus* Bertol.	0	0	0	0	0	0	0	3	0	0	3
*Erigeron strigosus* Muhl. Ex Willd.	0	0	3	8	9	0	0	0	0	0	20
*Eupatorium hyssopifolium* L.	0	0	0	0	0	0	1	0	0	0	1
*Euphorbia discoidalis* Chapm.	0	0	0	0	0	0	0	9	0	0	9
*Galactia regularis* (L.) Britton, Sterns & Poggenb.	0	0	0	0	0	0	3	0	0	0	3
*Gelsemium sempervirens* (L.) J. St.‐Hil.	43	0	0	0	0	0	0	0	0	0	43
*Helianthus angustifolius* L.	0	0	0	0	0	0	0	0	179	37	216
*Helianthus hirsutus* Raf.	0	0	0	0	0	0	5	0	0	0	5
*Helianthus strumosus* L.	0	0	0	0	6	1	1	4	0	0	12
*Hibiscus aculeatus* Walter	0	0	0	0	0	1	8	0	0	0	9
*Hieracium gronovii* L.	0	0	0	0	0	0	0	1	0	0	1
*Hypericum cistifolium* Lam.?	0	0	0	0	0	2	0	0	0	0	2
*Ipomoea pandurata* (L.) G. Mey.	0	0	0	22	14	3	9	0	0	0	48
*Lespedeza hirta* (L.) Hornem.	0	0	0	3	3	0	0	9	0	0	15
*Liatris gracilis* Pursh	0	0	0	0	0	0	0	0	1	0	1
*Liatris pilosa* (Aiton) Willd.	0	0	0	0	0	0	0	0	1	0	1
*Lobelia puberula* Michx.	0	0	0	0	0	0	0	2	1	0	3
*Ludwigia alternifolia* L.	0	0	0	0	0	0	1	0	0	0	1
*Mimosa quadrivalvis* L.	0	0	15	47	28	0	3	0	0	0	93
*Mitracarpus hirtus* (L.) DC.	0	0	0	0	0	0	0	1	0	0	1
*Oxalis dillenii* Jacq.	4	8	3	0	0	0	0	0	0	0	15
*Passiflora incarnata* L.	0	0	0	1	2	3	0	0	0	0	6
*Piriqueta cistoides* (L.) Griseb.	0	0	0	3	2	0	1	0	0	0	6
*Pityopsis aspera* (Shuttlw. ex Small) Small	1	0	0	2	1	0	0	29	29	17	79
*Polygala nana* (Michx.) DC.	0	2	3	0	0	0	0	0	0	0	5
*Polygala polygama* Walter	0	2	15	17	0	0	0	0	0	0	34
*Prenanthes serpentaria* Pursh	0	0	0	0	0	0	0	0	1	0	1
*Rhexia mariana* L.	0	0	0	0	0	0	2	0	0	0	2
*Rhus copallinum* L.	0	0	0	0	0	0	104	0	0	0	104
*Rhynchosia tomentosa* (L.) Hook & Arn.	0	0	0	7	3	0	0	0	0	0	10
*Rubus cuneifolius* Pursh	0	2	93	11	0	0	0	0	0	0	106
*Rubus flagellaris* Willd.	0	3	0	0	0	0	0	0	0	0	3
*Rudbeckia hirta* L.	0	0	0	0	2	0	0	0	0	0	2
*Sabatia angularis* (L.) Pursh	0	0	0	0	0	5	3	0	0	0	8
*Salvia azurea* Michx. ex Lam.	0	0	0	0	0	0	0	0	3	0	3
*Scutellaria incana* Biehler	0	0	0	0	0	0	2	2	0	0	4
*Scutellaria integrifolia* L.	0	0	0	0	0	0	0	1	0	0	1
*Scutellaria multiglandulosa* (Kearney) Small ex Harper	0	0	0	0	0	0	0	1	0	0	1
*Sericocarpus tortifolius* (Michx.) Nees	0	0	0	0	0	0	0	27	10	0	37
*Silphium asteriscus* L.	0	0	0	0	26	12	6	0	0	0	44
*Solidago altissima* L.	0	0	0	0	0	7	0	0	25	0	32
*Solidago arguta* Aiton	0	0	0	0	0	0	0	35	0	0	35
*Solidago odora* Aiton	0	0	0	1	0	0	0	0	7	0	8
*Solidago petiolaris* Aiton	0	0	0	0	0	0	0	0	24	1	25
*Stylisma patens* (Desr.) Myint	0	0	0	0	21	0	0	0	0	0	21
*Symphyotrichum dumosum* (L.) G.L. Nesom	0	0	0	0	0	0	0	0	2	1	3
*Tephrosia virginiana* (L.) Pers.	0	0	42	99	0	0	0	0	0	0	141
*Trichostema dichotomum* L.	0	0	0	0	0	0	0	16	0	0	16
*Triodanis perfoliata* (L.) Nieuwl.	0	0	2	0	0	0	0	0	0	0	2
*Vaccinium stamineum* L.	0	0	3	0	0	0	0	0	0	0	3
*Vaccinium virgatum* Aiton	0	36	0	0	0	0	0	0	0	0	36
*Verbesina aristata* (Elliot) A. Heller	0	0	0	0	5	0	0	0	0	0	5
*Vernonia angustifolia* Michx.	0	0	0	0	1	12	3	4	0	0	20
Total abundance	49	241	212	235	213	48	155	190	398	128	1869
Total number of species	4	10	15	17	17	11	16	23	16	8	77

## References

[ece310450-bib-0001] Adedoja, O. A. , Crandall, R. M. , & Mallinger, R. E. (2022). Season of prescribed burns and management of an early successional species affect flower density and pollinator activity in a pine savanna ecosystem. PeerJ, 10, e14377. 10.7717/peerj.14377 36389407 PMC9661972

[ece310450-bib-0002] Bond, W. J. , & Parr, C. L. (2010). Beyond the forest edge: Ecology, diversity and conservation of the grassy biomes. Biological Conservation, 143(10), 2395–2404. 10.1016/j.biocon.2009.12.012

[ece310450-bib-0003] Brown, J. , York, A. , Christie, F. , & McCarthy, M. (2017). Effects of fire on pollinators and pollination. Journal of Applied Ecology, 54, 313–322.

[ece310450-bib-0004] Cáceres, M. D. , & Legendre, P. (2009). Associations between species and groups of sites: Indices and statistical inference. Ecology, 90(12), 3566–3574. 10.1890/08-1823.1 20120823

[ece310450-bib-0005] Carbone, L. M. , Tavella, J. , Pausas, J. G. , & Aguilar, R. (2019). A global synthesis of fire effects on pollinators. Global Ecology and Biogeography, 28(10), 1487–1498. 10.1111/geb.12939

[ece310450-bib-0006] Cayuela, L. , & Gotelli, N. J. (2014). rareNMtests: Ecological and Biogeographical Null Model Tests for Comparing Rarefaction Curves. R Package v1. Vienna: The R Foundation for Statistical Computing.

[ece310450-bib-0007] Danforth, B. N. , Minckley, R. L. , Neff, J. L. , & Fawcett, F. (2019). The solitary bees: Biology, evolution, conservation. Princeton University Press.

[ece310450-bib-0008] Engle, D. M. , Palmer, M. W. , Crockett, J. S. , Mitchell, R. L. , & Stevens, R. (2000). Influence of late season fire on early successional vegetation of an Oklahoma prairie. Journal of Vegetation Science, 11(1), 135–144. 10.2307/3236785

[ece310450-bib-0009] Folkerts, G. W. , Deyrup, M. A. , & Sisson, D. (1993). Arthropods associated with xeric longleaf pine habitats in the southeastern United States: A brief overview . Paper presented at the Proceedings of the Tall Timbers Fire Ecology Conference.

[ece310450-bib-0010] Gibbs, J. (2011). Revision of the metallic Lasioglossum (Dialictus) of eastern North America (Hymenoptera: Halictidae: Halictini). Zootaxa, 3073, 1–216.

[ece310450-bib-0011] Glitzenstein, J. S. (2003). Long‐Term Seasonal Burning at the St. Marks National Wildlife Refuge, North Florida: Changes in the Sandhill Plots After 23 Years. *Proceedings of the 2nd International Wildland Fire Ecology and Fire Management Congress*.

[ece310450-bib-0012] Guyette, R. P. , Stambaugh, M. C. , Dey, D. C. , & Muzika, R.‐M. (2012). Predicting fire frequency with chemistry and climate. Ecosystems, 15(2), 322–335. 10.1007/s10021-011-9512-0

[ece310450-bib-0013] He, T. , Lamont, B. B. , & Pausas, J. G. (2019). Fire as a key driver of Earth's biodiversity. Biological Reviews, 94(6), 1983–2010. 10.1111/brv.12544 31298472

[ece310450-bib-0014] Hiers, J. K. , Wyatt, R. , & Mitchell, R. J. (2000). The effects of fire regime on legume reproduction in longleaf pine savannas: Is a season selective? Oecologia, 125(4), 521–530. 10.1007/s004420000469 28547222

[ece310450-bib-0015] Hothorn, T. , Bretz, F. , Westfall, P. , Heiberger, R. M. , Schuetzenmeister, A. , Scheibe, S. , & Hothorn, M. T. (2016). *Package ‘multcomp’*. *Simultaneous inference in general parametric models*. *Project for Statistical Computing*, *Vienna*, *Austria* .

[ece310450-bib-0016] Jue, D. K. , Merwin, A. C. , Jue, S. S. , McElveen, D. , & Inouye, B. D. (2022). Effects of frequency and season of fire on a metapopulation of an imperiled butterfly in a longleaf pine forest. Conservation Science and Practice, 4(8), e12739. 10.1111/csp2.12739

[ece310450-bib-0017] Krings, A. , Szakacs, A. D. , & Hyland, E. G. (2023). Remnants of the “Grande Savane?” insights from soil organic matter at two sites in the Deep River Triassic Basin of North Carolina. Castanea, 87(2), 244–267, 224.

[ece310450-bib-0018] Lewis, C. E. (1964). *Forage response to month of burning*: Southeastern Forest Experiment Station, US Department of Agriculture.

[ece310450-bib-0019] Lewis, C. E. , & Harshbarger, T. J. (1976). Shrub and herbaceous vegetation after 20 years of prescribed burning in the South Carolina coastal plain. Journal of Range Management, 29, 13–18.

[ece310450-bib-0020] McCune, B. , & Mefford, M. J. (2011). PC‐ORD. Multivariate Analysis of Ecological Data, Version 6. Gleneden Beach: MjM Software.

[ece310450-bib-0021] McLauchlan, K. K. , Higuera, P. E. , Miesel, J. , Rogers, B. M. , Schweitzer, J. , Shuman, J. K. , Tepley, A. J. , Varner, J. M. , Veblen, T. T. , Adalsteinsson, S. A. , Balch, J. K. , Baker, P. , Batllori, E. , Bigio, E. , Brando, P. , Cattau, M. , Chipman, M. L. , Coen, J. , Crandall, R. , … Watts, A. C. (2020). Fire as a fundamental ecological process: Research advances and frontiers. Journal of Ecology, 108, 2047–2069.

[ece310450-bib-0022] Mitchell, T. B. (1960). *Bees of the Eastern United States Volume 1*: The North Carolina Agricultural Experiment Station.

[ece310450-bib-0023] Mitchell, T. B. (1962). *Bees of the Eastern United States Volume 2*. The North Carolina Agriculture Experiment Station.

[ece310450-bib-0024] Palmer, W. E. , & Sisson, D. C. (2017). Tall timbers bobwhite quail management handbook. Tall Timbers Press.

[ece310450-bib-0025] Platt, W. J. , Evans, G. W. , & Davis, M. M. (1988). Effects of fire season on flowering of forbs and shrubs in longleaf pine forests. Oecologia, 76(3), 353–363. 10.1007/BF00377029 28312014

[ece310450-bib-0026] Ponisio, L. C. , Wilkin, K. , M'Gonigle, L. K. , Kulhanek, K. , Cook, L. , Thorp, R. , Griswold, T. , & Kremen, C. (2016). Pyrodiversity begets plant–pollinator community diversity. Global Change Biology, 22(5), 1794–1808. 10.1111/gcb.13236 26929389

[ece310450-bib-0027] R Core Team . (2022). R: A language and environment for statistical computing. R Foundation for Statistical Computing, Vienna, Austria.

[ece310450-bib-0028] Robertson, K. M. , & Hmielowski, T. L. (2014). Effects of fire frequency and season on resprouting of woody plants in southeastern US pine‐grassland communities. Oecologia, 174, 765–776. 24213629 10.1007/s00442-013-2823-4

[ece310450-bib-0029] Robertson, K. M. , & Ostertag, T. E. (2007). Effects of land use on fuel characteristics and fire behavior in pinelands of Southwest Georgia. Tall Timbers Fire Ecology Conference Proceedings, 23, 181–191.

[ece310450-bib-0030] Ryan, K. C. , Knapp, E. E. , & Varner, J. M. (2013). Prescribed fire in North American forests and woodlands: History, current practice, and challenges. Frontiers in Ecology and the Environment, 11(s1), e15–e24. 10.1890/120329

[ece310450-bib-0031] Smith, M. D. , van Wilgen, B. W. , Burns, C. E. , Govender, N. , Potgieter, A. L. , Andelman, S. , Biggs, H. C. , Botha, J. , & Trollope, W. S. (2013). Long‐term effects of fire frequency and season on herbaceous vegetation in savannas of the Kruger National Park, South Africa. Journal of Plant Ecology, 6(1), 71–83.

[ece310450-bib-0033] Towne, G. , & Owensby, C. (1984). Long‐term effects of annual burning at different dates in ungrazed Kansas tallgrass prairie. Journal of Range Management, 37(5), 392–397.

[ece310450-bib-0034] Ulyshen, M. D. , Hiers, J. K. , Pokswinksi, S. M. , & Fair, C. (2022). Pyrodiversity promotes pollinator diversity in a fire‐adapted landscape. Frontiers in Ecology and the Environment, 20(2), 78–83. 10.1002/fee.2436

[ece310450-bib-0035] Van Wyk, P. (1971). Veld burning in the Kruger National Park: An interim report of some aspects of research . Paper presented at the Proceedings of the Tall Timbers Fire Ecology Conference.

[ece310450-bib-0036] Veldman, J. W. , Buisson, E. , Durigan, G. , Fernandes, G. W. , Le Stradic, S. , Mahy, G. , Negreiros, D. , Overbeck, G. E. , Veldman, R. G. , Zaloumis, N. P. , Putz, F. E. , & Bond, W. J. (2015). Toward an old‐growth concept for grasslands, savannas, and woodlands. Frontiers in Ecology and the Environment, 13(3), 154–162. 10.1890/140270

[ece310450-bib-0037] Waldrop, T. A. , White, D. L. , & Jones, S. M. (1992). Fire regimes for pine‐grassland communities in the southeastern United States. Forest Ecology and Management, 47, 195–210.

